# Psychometric properties of the Brief Problem Monitor (BPM) in children with internalizing symptoms: examining baseline data from a national randomized controlled intervention study

**DOI:** 10.1186/s40359-021-00689-1

**Published:** 2021-11-27

**Authors:** Marit Løtveit Pedersen, Thomas Jozefiak, Anne Mari Sund, Solveig Holen, Simon-Peter Neumer, Kristin D. Martinsen, Lene Mari P. Rasmussen, Joshua Patras, Stian Lydersen

**Affiliations:** 1 Regional Center for Child and Youth Mental Health and Child Welfare, Department of Mental Health, Norwegian University of Science and Technology, Trondheim, Norway; 2 Department of Child and Adolescent Psychiatry, St. Olavs University Hospital, Trondheim, Norway; 3 Centre for Child and Adolescent Mental Health, RBUP East and South, Oslo, Norway; 4 RKBU – North, Health Sciences Faculty, UiT The Arctic University of Norway, Tromsø, Norway

**Keywords:** Brief Problem Monitor, BPM-P, BPM-T, Internalizing problems, Anxiety, Depression, Children, Psychometric properties, Confirmatory factor analysis

## Abstract

**Background:**

Prevention is essential to reduce the development of symptomology among children and adolescents into disorders, thereby improving public health and reducing costs. Therefore, easily administered screening and early assessment methods with good reliability and validity are necessary to effectively identify children’s functioning and how these develop. The Brief Problem Monitor (BPM) is an instrument designed for this purpose. This study examined the psychometric properties of the Norwegian version of the BPM parent (BPM-P) and teacher (BPM-T) versions, including internal reliability and construct validity at assessing children with internalizing problems.

**Methods:**

Baseline data were collected from a national randomized controlled intervention study. Children aged 8–12 years with self-reported symptoms of anxiety and/or depression with one standard deviation above a chosen population’s mean were included in this study. Teachers (n = 750) and parents (n = 596) rated children using the BPM-T and BPM-P, respectively. Internal consistency was measured using Cronbach’s alpha, and multi-informant agreement between the BPM-P and BPM-T was measured using Spearman’s correlations. Construct validity was assessed via confirmatory factor analysis.

**Results:**

Internal consistency was good throughout all domains for both the BPM-P and BPM-T, with a Cronbach’s alpha ranging from .763 to .878. Multi-informant agreement between the parents and the teacher was moderate on the externalizing, attention, and total scales and low on the internalizing scale. The model fit for the three-factor structure of the BPM was excellent for the BPM-P and good for the BPM-T.

**Conclusions:**

Internal consistency was good, and the original three-factor solution of the BPM-P and BPM-T was confirmed based on our sample of school children at-risk for emotional problems. These promising results indicate that the BPM may be a valid short assessment tool for measuring attentional, behavioral, and internalizing problems in children.

*Trial registration* in Clinical Trials: NCT02340637; June 12, 2014.

## Background

Symptoms of anxiety and depression [internalizing problems] are among the most common psychological difficulties diagnosed in children and adolescents. Approximately seven percent of children from population-based samples in Norway present symptoms that are compatible with a mental disorder [1, 2]. Similarly, international studies have found a prevalence rate of mental health disorders from 7 to 23% [3–6]. Anxiety and depression often co-occur, and anxiety often precedes depression [7, 8]. Depressive problems affect youth negatively in different life domains (e.g., lower academic achievement, more peer and family problems) [4, 9, 10], and anxious youth are at greater risk for absenteeism, academic underachievement, low social acceptance, and impaired psychosocial functioning [11–13]. Previous research also indicates that children with symptoms of anxiety and/or depression clearly experience a reduction in their daily functioning, even though they do not qualify for a full diagnosis [14, 15]. Such negative outcomes can cause serious health consequences and costs, for the youth, his or her family, and the society at large [16]. Thus, it is important to prevent the development of anxiety and depression in children and adolescents. Having valid and reliable instruments to identify these children is, therefore, of utmost importance.

Both in research and clinical care, assessments of intervention progress and outcome that are quick, easily administered, valid, and reliable are needed [17] so that response to the intervention and possible adjustments to the intervention processes can be applied. Advantageously, the assessment method is suitable for children with comorbid conditions (e.g., anxiety, depression, conduct problems) and various informants. To be useful, a short survey should adequately assess progress and outcomes, accommodate comorbidity, take little time to administer, and show satisfactory psychometric properties. *The Brief Problem Monitor* (BPM) instrument is designed for this purpose [18]. This measure provides a uniform problem scale to assess attentional, behavioral, and internalizing problems in children and adolescents aged from 6 to 18 years. The BPM forms are developed from the longer corresponding versions of the ASEBA *Child Behavior Checklist for those aged *6–18* years* (CBCL/6–18), *Teachers’ Report Form for children those aged *6–18 (TRF), and *Youth Self-report for those aged *11–18 *years* (YSR) [19]. The ASEBA long forms are widely used instruments for clinical and research psychopathology. They have for decades provided information from various informants and shown good psychometric properties in studies conducted in different countries [19, 20]. To date, the literature supporting the psychometric properties of the Brief Problem Monitor (BPM) measures is scarce. In the present study, we examined the psychometric properties of the Norwegian versions of the BPM for parents (BPM-P) and teachers (BPM-T).

### Development of the BPM survey

The development of the BPM started with Chorpita and colleagues’ Brief Problem Checklist interview (BPC), a brief 12-item index derived from YSR and CBCL, meant to be an easily administered, time-saving, and clinically relevant measure [17]. The index included internalizing (INT) and externalizing problems (EXT). Despite the reduced number of items in the subscales, internal consistency, test–retest reliability, and convergent and discriminant validity were considered good [17].

Achenbach et al. [18] expanded the index further and added an additional third scale in the BPM, assessing attention and hyperactive symptoms (ATT). Achenbach also developed an assessment for teachers based on the TRF. The final version of the BPM consists of one form for parents (BPM-P; 19 items), one form for adolescents (BPM-Y; 19 items), and one form for teachers (BPM-T; 18 items) [18]. The items are distributed on three subscales: attention/hyperactivity problems (6 items), externalizing problems (6 items in BPM-T and 7 items in BPM-P and BMP-Y), and internalizing problems (6 items).

### Psychometric properties of BPM

Studies of the 2001 version of the ASEBA long forms were used to analyze the psychometric properties of the American versions of the BPM [18, 19]. In a US sample of youths, the CBCL- and TRF-forms showed excellent internal consistency [19]. Test–retest (8–16 days apart) yielded similar results. They also reported good criterion-related validity to differentiate between children with and without a diagnosis.

Apart from Achenbach and colleagues [19], there are, to our knowledge, four published studies evaluating the psychometric properties of the BPM forms, see Table 1 [21–24].

**Table 1:** Psychometric characteristics of BPM reported in four studies

Study (country)			BPM-P				BPM-T			
			ATT	INT	EXT	Total	ATT	INT	EXT	Total
Richter [21] (Norway)	Type of population		General population				Stratified random subsample			
	Age range (mean)		6–16 (10.6)				6–13 (9.4)			
	Reliability	*Internal consistency* (*Cronbach’s alpha*)	.77	.70	.76	.93	.88	.71	.87	.96
Piper et al. [23] (USA/Canada)	Type of population		Convenience sample							
	Age range		6–18 (mean age 11.5)							
	Reliability	*Internal consistency* (*Cronbach’s alpha*)	.87	.79	.86	.91	BPM-T not conducted			
Penelo et al. [22] (Spain)	Type of population		General population							
	Age range		6–8							
	Reliability	*Internal consistency* (*McDonald’s Omega*)	.92	.83	.89	.93	BPM-T not conducted			
	Validity	*Construct RMSEA*	.040^a^/.052^b^/.057^c^				“			
		*CFI*	.968^a^/.930^b^/.919^c^				“			
		*TLI*	.963^a^/.920^b^/.907^c^				“			
Rodenacker et al. [24] (Germany)	Type of population		Clinical/general population				Clinical/general population			
	Age range		Mean age 11.5/12.3				Mean age 11.5/12.3			
	Reliability	*Internal consistency* (*Cronbach’s alpha*)	.83/.81	.72/.66	.81/.73	.83 /.83	.76/.85	.76/.74	.86/.87	.81/.87
	Validity	*Construct RMSEA*	.077/.045				.119/.102			
		*CFI*	.920/.950				.890/.929			
		*TLI*	.906/.941				.872/.918			

BPM-P = Brief Problem Monitor–Parents; BPM-T = Brief Problem Monitor–Teacher; ATT = attention; EXT = externalizing problems; INT = internalizing problems; Total = total problems (ATT + EXT + INT). Validity: Robust weighted least squares estimator: RMSEA = root mean square error of approximation, CFI = comparative fit index, TLI = Tucker Lewis index. Age of the sample: ^a^ = 6-year-old, ^b^ = 7-year-old, ^c^ = 8-year-old

References: Richter [21], Piper et al. [23], Penelo et al. [22], Rodenacker et al. [24]

According to a systematic review of Scandinavian studies on the psychometric properties of the BPM [25], only one study was found [21]. This study by Richter [21] included BPM-P, BPM-T, and BPM-Y in a Norwegian population sample of children ranging in age from 6 to 16 years. The study reported excellent internal consistency for the total scale for all versions according to the European Federation of Psychologists’ Association’s guidelines (EFPA) [26]. The internal consistency of the internalizing subscale was reported to be adequate for BPM-P and BPM-T. For the remaining two subscales, attention and externalizing problems, the internal consistency was adequate on BPM-P and good on BPM-T. The study also reported good construct and content (convergent) validity.

The second study was performed with an American/Canadian sample; caregivers of children/youths aged 6–18 years completed the CBCL/6–18 online [23]. The 19 items of the BPM-P were analyzed. The internal consistency for the BPM-P total scale was excellent, the attention and externalizing subscales were good, and the internalizing subscale was adequate. The correlation between the full-length CBCL/6–18 and the shorter BPM-P was considered high for the total score and the subscales. BPM-P was sensitive and could identify behavioral and emotional problems among children whose parents reported a psychiatric diagnosis (ADHD, depression, anxiety, autism spectrum disorders, etc.) when compared to the group that had not been diagnosed. However, the study was limited to caregivers, and the findings supported that additional information from other sources, for example, teachers, should be obtained.

The third study was conducted in a community sample of Spanish children aged from 6 to 8 years, where parents answered the CBCL/6–18. Nineteen items of the BPM-P were examined [22]. Internal consistency was good. The subscales showed higher values for attention problems and lower for internalizing problems. The concurrent validity was high with a significant correlation between BPM-P and CBCL/6–18. Construct validity, investigated by confirmatory factor analysis (CFA), showed that the 3-factor model (attention, externalizing, internalizing) was adequate.

The fourth study was conducted in a clinical and a general population-based sample from Germany. Children, parents, and teachers answered the BPM based on the long version of ASEBA from 1991 [24]. Two items on the attention scale were not present (“fails to finish things he/she starts” and “inattentive or easily distracted”), leaving 17 items. The internal consistency was considered adequate-to-good for most of the subscales and the total scale regarding BPM-P and BPM-T in both samples. The subscale of internalizing problems showed inadequate consistency in the general population sample of parents. BPM-P indicated an acceptable three-factor model fit in the clinical sample and an excellent model fit in the general population sample. Regarding the BPM-T, the teacher-version did not have a satisfactory model fit in the clinical or the general population sample.

### Multiple informant differences

A meta-analysis that evaluated the validity of multiple informants assessing child and adolescent mental health problems in 341 studies from 1989 to 2014 reported low-to-moderate cross-informant correspondence (mean internalizing: correlation 0.25; externalizing 0.30; mean overall 0.28) [27]. The meta-analysis indicated higher levels of correspondence when problems were easy to observe (externalizing behavior vs. internalizing problems), and informants came from the same setting (mother and father vs. parent and teacher). This is similar to studies evaluating BPC/BPM/ASEBA long forms, where the cross-informant agreement was low (0.22, 0.31, and 0.19 for the internalizing, externalizing, and total scales, respectively) between *child and parent* [17]. Achenbach [28] found a parent–child correlation of 0.25 and a higher cross-informant correlation for externalizing than internalizing problems across diverse types of cross-informant pairs.

Correlations between *parents and teachers* are somewhat higher in these studies—ranging from 0.38 to 0.44 for the attention scale, 0.32– 0.35 for the externalizing scale, 0.21 for the internalizing scale, and 0.33 for the total scale [19, 29]. All these findings are in accordance with Achenbach’s multi-informant assessment approach, i.e., that high cross-informant agreement regarding psychological problems/mental health symptoms is not expected because mental health problems are perceived differently from different perspectives. Furthermore, the problems may only be present in certain settings.

To summarize, studies of the BPM are scarce and previous studies have been performed in clinical or population samples. Children with self-reported symptoms of anxiety and depression are an understudied at-risk population, and there is a call for valid, reliable, and easily administered instruments to assess these symptoms as early as possible. Moreover, the psychometric properties of translated versions of the BPM should be evaluated. The cultural norms and differences between countries are important and could influence the results and usefulness of the scale. The only study from Scandinavia [21] was population-based and did not include any factor analysis of the BPM. Furthermore, the only study including two types of informants and a confirmatory factor analysis did not contain all the BPM`s items [24]. This study aimed to evaluate the psychometric properties of the Norwegian version of the BPM in young children (aged 8–12 years) at-risk of developing anxiety and depression, based on reports from both parents and teachers.

## Methods

### Procedure

The current study used baseline data from a national randomized controlled intervention study [30] investigating the effectiveness of the intervention *EMOTION, Coping Kids Managing Anxiety and Depression* [31] for children aged 8–12 years. The EMOTION intervention aims to reduce symptoms of anxiety and depression and the likelihood of developing later disorders.

Primary schools from rural, suburban, and urban areas in Norway volunteered to participate in the study. The children in the eligible grades (8–12 years of age) and their parents were informed through age-appropriate information in class and at parental meetings about the study both in writing and orally. Children who considered themselves more anxious or sadder than their peers were invited to participate in the screening procedure. Participation required expressed interest from the child and written parental consent; teachers were also informed about the study. Data were collected electronically from 2014 until 2017 with new children entering every semester. For further information about the randomized controlled trial, see Patras and colleagues [30].

### Participants

Children (n = 1692) from 36 primary schools were screened using web-based questionnaires for self-reported symptoms of anxiety and depression, using the Multidimensional Anxiety Scale for Children—MASC-C [32] and the Mood and Feelings Questionnaire-Short Version—SMFQ [33].

Children who reported symptom levels of at least one standard deviation above the population-based mean on measures of anxiety and/or depression were invited to participate in the study (n = 873). These cut-offs were based on national and international studies in the relevant age group [34–36]. Seven of the invited children, who were not expected to benefit from the intervention (having developmental delays, autism, severe behavioral disturbance), were excluded from the study. Due to a lack of resources (i.e., lack of group leaders to implement the intervention), 71 children were also randomly excluded.

Web-based questionnaires were sent to the participating children’s parents and teachers approximately two weeks after their screening. Although both parents were encouraged to participate, participation was voluntary; however, the children were invited regardless of whether their parents had answered the questionnaires. The child’s primary teacher answered questionnaires about the child. The parents and teachers rated the children on matters concerning attention/hyperactivity, internalizing problems, and externalizing problems now or within the last two weeks, using BPM-P and BPM-T, respectively.

Of the included children, 750 (n = 435, 58% girls) students had a teacher response and were included in the current study. Grade level was used as a proxy for age: Third to sixth grade represented 8–12 years of age. Approximately 4% of the participating children were in the third grade, 36.1% in fourth, 46.1% in fifth, and 13.7% in sixth. Only one parental response per child was analyzed in the present study; 596 children had a response from one of the parents (482 mothers, 80.6%).

### Instruments

*Brief Problem Monitor (BPM).* The BPM-P (19 items) and BPM-T (18 items) has an age range from 6 to 18 years. The instruments include three subscales with six items each: ATT, INT, and EXT. The extra question on the parent version is about disobedient behavior at home. The ATT subscale contains questions like whether the child “can’t concentrate or pay attention for long;” or “can’t sit still, restless or hyperactive.” Within the EXT subscale, there are questions on whether the child “argues a lot” or “has temper tantrums or a hot temper.” In the INT subscale, the questions ask if the child is “feeling too fearful or anxious” or “unhappy, sad, or depressed.” The items are rated over user-selected rating periods (e.g., 5,7,14,30, and 45 days), and are supposed to describe the child, on a scale ranging from 0 to 2 (0 = *not true*, 1 = *somewhat true*, or 2 = *very true*).

The Norwegian versions of the CBCL, TRF, and YSR were translated and published in 1986/1988, 1993, and 2002, respectively [37]. The Norwegian version of the BPM was based on these translations.

*The Multidimensional Anxiety Scale for Children–Child (MASC-C).* In this study, children were included using the MASC-C [32] to assess anxiety symptoms, a 39-item self-report questionnaire that assesses anxiety symptoms in children and adolescents aged 8–19 years based on the past two weeks.

The MASC-C has shown favorable psychometric properties both internationally and in Norway [32, 38–40]. The internal consistency of self-reported symptoms of anxiety at baseline in the current study was good, with a Cronbach’s alpha of 0.84.

*The Mood and Feelings Questionnaire-short version (SMFQ).* To assess depressive symptoms, SMFQ [33], a 13-items questionnaire targeting children from 8 to 18 years, was used. The SMFQ assesses cognitive, affective, and behavioral-related symptoms of depression during the last two weeks.

Previous studies indicated good psychometric properties for the Norwegian version of the SMFQ [41, 42]. In this study, the internal consistency of the scale was good, with a Cronbach’s alpha = 0.80 at baseline.

### Statistics analysis

For the children who had teacher reports (n = 750), 154 parental answers were missing. For the participating parents (n = 596) and the teachers, no missing items were reported.

Reliability was measured by internal consistency (Cronbach’s alpha) of the subscales and the total problem scale. According to EFPA [26], the internal consistency is considered excellent if Cronbach’s alpha > 0.90, good between 0.80 and 0.90, adequate between 0.70 and 0.79, and inadequate when < 0.70.

The items on BPM-P and BPM-T have only three ordinal response categories (*not true, somewhat true, very true*). The responses were not normally distributed. To assess multi-informant agreement between the subscales on BPM-P and BPM-T, we used Spearman’s correlation coefficient. According to Cohen [43], correlation coefficients were considered low between 0.10 and 0.29, moderate between 0.30 and 0.49, and high for 0.50 and above.

To assess construct validity, we conducted a CFA for ordinal categorical variables to confirm the conceptual three-factor model with the three subscales of the BPM-P and BPM-T. Weighted least square estimator with robust standard errors and mean- and variance-adjusted chi-square test statistics (WLSMV) were used as an estimator, owing to the ordinal structure of the data. The robust weighted least squares (WLSMV) estimator with Delta parameterization is recommended for the analysis of skewed categorical data [44]. In this method, error points are accounted for, and the items are interpreted as observable indicators of the non-observable (latent) factor to which they belong [45]. The following goodness-of-fit indices were used: RMSEA, CFI, and TLI. The indices were recommended by Brown [46]: RMSEA < 0.08 and CFI and TLI > 0.90 were considered acceptable fit, and RMSEA < 0.06 and CFI and TLI > 0.95 indicate excellent fit. The factor loadings for each item on the associated subscale were evaluated according to Tabachnick and Fidell’s [47] suggestions, where a rating of 0.71 is considered excellent, 0.63 very good, 0.55 good, 0.45 fair, and 0.32 and lower poor.

Two-sided *p*-values < 0.05 were regarded as significant, and we reported 95% confidence intervals (CI) where relevant. CFA analyses were conducted using Mplus 8 [48]. Other analyses were conducted using SPSS 26.0 (IBM, Armonk, NY, USA).

## Results

### Descriptive statistics for the BPM-P and BPM-T subscales and total scores

Table 2 shows the total mean scores and standard deviation for the different subscales and the total score on BPM-P and BPM-T.

**Table 2:** Mean scores and standard deviation for the BPM-P and BPM-T

Domains	BPM-P (n = 596)		BPM-T (n = 750)	
	Number of items	Mean (SD)	Number of items	Mean (SD)
ATT	6	2.92 (2.88)	6	3.09 (3.27)
EXT	7	2.26 (2.50)	6	1.30 (2.07)
INT	6	2.58 (2.49)	6	2.57 (2.61)
Total	19	7.76 (6.15)	18	6.96 (6.12)

BPM-P = Brief Problem Monitor–Parents; BPM-T = Brief Problem Monitor–Teacher; ATT = attention; EXT = externalizing problems; INT = internalizing problems; Total = total problems (ATT + EXT + INT); SD = standard deviation

### Reliability

The BPM-P showed good internal consistency on attention problems (α = 0.834), on externalizing problems (α = 0.805), and the total problems scale (α = 0.871); and adequate internal consistency on internalizing problems (α = 0.763). The BPM-T showed good internal consistency on all the subscales and total problems scale: attention (0.878), externalizing problems (0.805), internalizing problems (0.818), and total problems (0.877).

### Multi-informant agreement

The correlations between BPM-P and BPM-T were moderate on the total problem scale (0.384) and the subscales of ATT (0.451) and EXT (0.391). The correlation was low on INT (0.290). See Table 3.

**Table 3:** Spearman’s correlations between BPM-P and BPM-T subscales and total score

BPM-T	BPM-P (n = 596)			
	ATT	EXT	INT	Total
ATT	.451**	.491**	.047	.325**
EXT	.255**	.391**	.089*	.270**
INT	.211**	.186**	.290**	.293**
Total	.409**	.315**	.181**	.384**

BPM-P = Brief Problem Monitor-parents; BPM-T = Brief Problem Monitor-teacher; ATT = attention; EXT = externalizing problems; INT = internalizing problems; Total = total problems (ATT + EXT + INT). *p < .05 (two-tailed), **p < .01 (two-tailed)

### Validity

#### Construct validity

The CFA analyses produced an excellent model fit for the BPM-P and a good model fit for the BPM-T for the three-factor model. See Table 4*.* The chi-square statistics were significant, as expected for a large sample size. The χ^2^/*df*-ratio was 2.6845 for the BPM-P and 6.6287 for the BPM-T; below 3 is commonly regarded as acceptable [46, 49, 50].

**Table 4:** Confirmatory factor analyses of the BPM-P and BPM-T: fit statistics for the model

Fit statistic	BPM-P	BPM-T
χ^2^/df	400.928/149 = 2.6845	875.361/132 = 6.6287
RMSEA	0.053 (CI 0.047–0.060)	0.087 (CI 0.081–0.092)
CFI	0.966	0.947
TLI	0.961	0.938

BPM-P = Brief Problem Monitor-parents, BPM-T = Brief Problem Monitor-teacher, Robust weighted least squares estimator, χ^2^/*df* = chi-square relative to its degree of freedom, RMSEA = root mean square error of approximation, CFI = comparative fit index, TLI = Tucker Lewis index

**Fig. 1:**
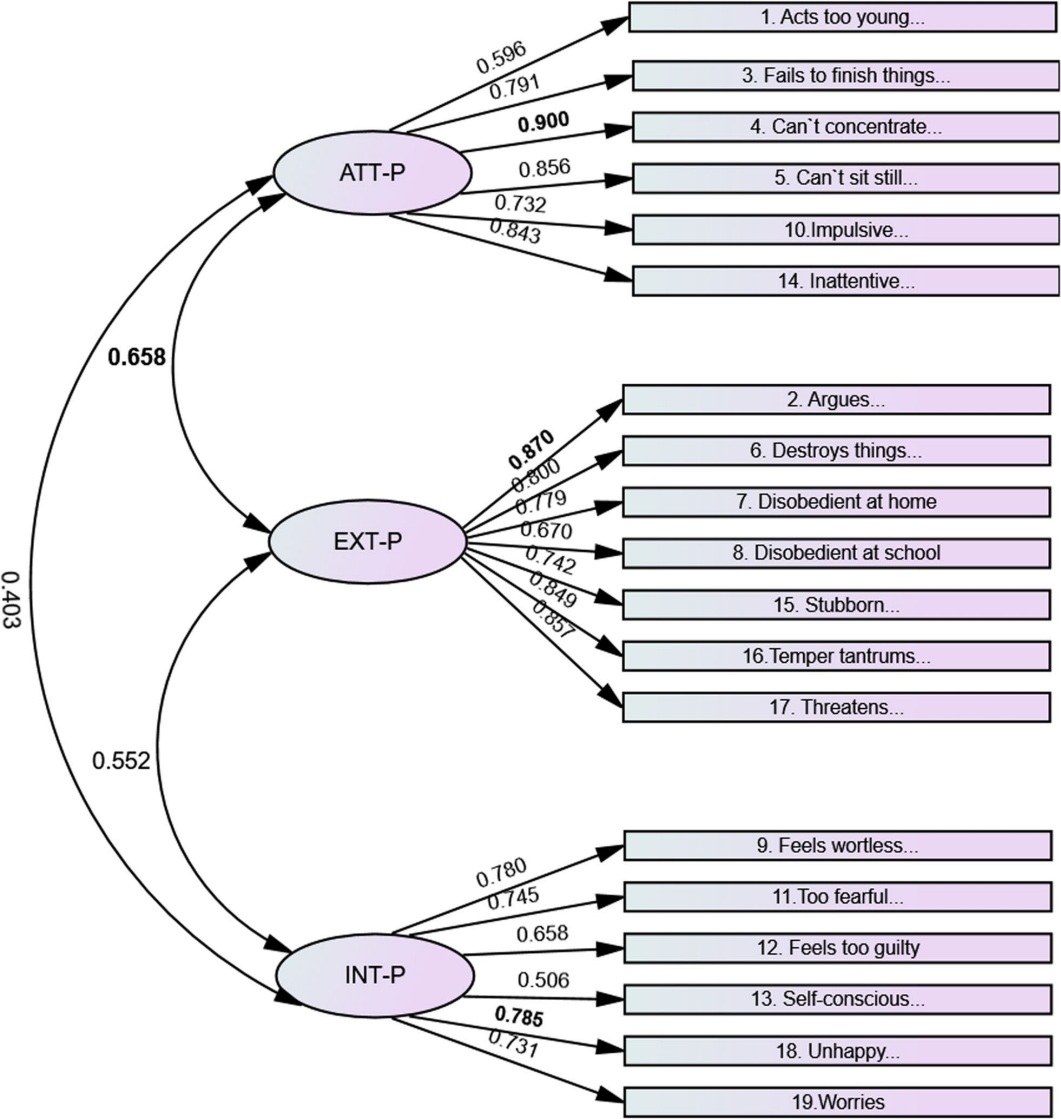
BPM-P. Standardized factor loadings and factor correlations. *Note*: BPM-P = Brief Problem Monitor-Parents. ATT = Attention; EXT = Externalizing problems; INT = Internalizing problems; P = parents. All the factors are significant at *p* < 0.01. In bold; highest correlation between factors, and items with the highest factor loading

**Fig. 2:**
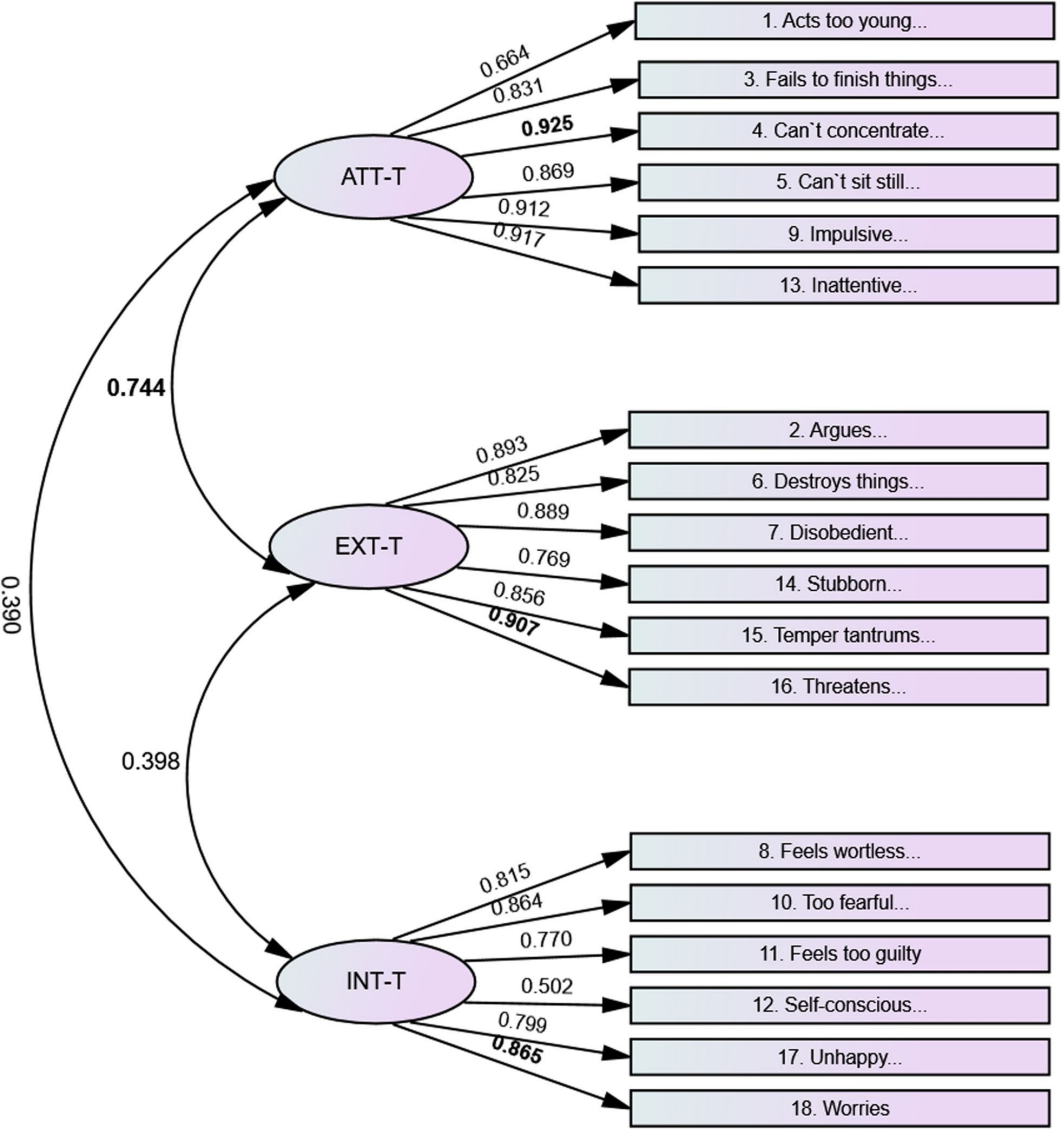
BPM-T. Standardized factor loadings and factor correlations. *Note*: BPM-T = Brief Problem Monitor-Teacher. ATT = Attention; EXT = Externalizing problems; INT = Internalizing problems; P = parents. All the factors are significant at *p* < 0.01. In bold; highest correlation between factors, and items with the highest factor loading

Further, all items on each subscale contributed significantly (*p* < 0.001) to the three latent constructs—EXT, ATT, and INT—with satisfactory factor loadings. See Figs. 1 and 2.

For the BPM-P, the factors that correlated highest were attention and externalizing problems (ATT–EXT = 0.658). See Fig. 1. The lowest factor correlation was between attention and internalizing problems (ATT–INT = 0.403). The items with the highest loading on the subscales were “Can’t concentrate; can’t pay attention for long” (ATT = 0.900); “Argues a lot” (EXT = 0.870); and “Unhappy, sad, or depressed” (INT = 0.785).

For the BPM-T, the factors that correlated highest were attention and externalizing problems (0.744). The lowest factor correlation was between attention and internalizing problems (0.390). The items with the highest loading on the subscales were “Can’t concentrate; can’t pay attention for long” (ATT = 0.925), “Threatens people” (EXT = 0.907), and “Worries” (INT = 0.865). See Fig. 2.

## Discussion

This is the first study to present the psychometric properties concerning both the reliability and construct validity of the Norwegian version of the BPM-P and BPM-T, used to assess schoolchildren with internalizing problems. The internal consistency for both versions was good. The multi-informant agreement was moderate to low. The model fit for the three-factor structure of the BPM was confirmed: excellent for the BPM-P and good for the BPM-T.

The internal consistency, assessed by Cronbach’s alpha, for the total scale and both the BPM-P and BPM-T were good, and the subscales generally showed good estimates. Hence, internal consistency was higher in the current study than in a representative sample from the general population in Norway [21]. In line with previous studies [21–24], this study also found higher values of attention problems than internalizing problems on both the BPM-P and BPM-T. Attention problems might be expressed through behaviors that are more visible to others, such as failing to finish tasks the child has started, inability to sit still, acting without thinking, etc. [51]. In contrast, internalizing problems exist more within the individual (e.g., feeling worthless, having worries, etc.), which are not so observable by others [52]. Children’s self-reports might be better suited to identify these problems, and triangulation of responses will create a better overall picture of children’s problem areas [27, 53].

Considering multi-informant perspectives from parents and teachers, we found moderate associations between corresponding subscales of attention, externalizing problems, and the total problem scale, and a weak association between the scorings of internalizing problems. Our findings corroborate previous research indicating that it is more common to agree on externalizing behavioral and attention problems than internalizing problems [19, 27–29]. It is important to underline that in child psychology, the associations between responses from different informants are expected to be low-to-moderate because mental health problems, per se*,* are perceived from different perspectives and in different environments. Thus, our results are in line with previous studies [19, 27–29].

Concerning the children in our high-scoring at-risk sample, our results showed a better model fit regarding both BPM-P and BPM-T compared to clinical and population-based samples [22, 24]. In the present study, all the items of the BPM were included in the analyses of construct validity, while in a German study, only 17 items were used [24]. The German study also showed that the teacher version did not have a satisfactory model fit in the clinical or the population sample. Our study, however, yielded an acceptable model fit for the teacher version. This indicates that in the school-based population studied, the complete version of the BPM can assess different problems in young school children from teachers’ perspectives as well. The results provide an opportunity for future use of the teacher version when examining children’s mental health problems to include significant school perspectives. However, more research is needed to confirm our findings. It is crucial to identify children with mental health issues early to prevent possible negative trajectories leading to anxiety and depression and reduced functioning in different life domains [4, 9, 12, 13]. Therefore, having available instruments that are easily administered, reliable and valid both for clinical and municipal health services are essential. It is also important to include different perspectives from different areas of the children’s lives, such as at home and school. Children can, for example, behave differently at home, where they feel safe. However, in school, which may have more unpredictable surroundings, the same child may struggle. Teachers can observe children’s behavior and well-being at school. The positive findings from our study are a much-needed supplement to the research on BPM in an understudied population of at-risk children.

Regarding the factor correlations on both BPM-P and BPM-T, attention and externalizing problems had the highest correlations, whereas attention and internalizing problems showed the lowest factor correlations. This result was not surprising given that externalizing issues may co-occur with attentional problems [51]. Children with internalizing problems may, however, often show avoidant behavior to their surroundings to cope with their fears [54], which may be more difficult to observe. Furthermore, the problems may be present at school and not at home or vice versa, which may make it even more difficult to identify. When exploring the factor loadings on the subscale of ATT, five of the six items showed excellent values. The exception was the item, “Acts too young for his/her age”; however, it was still considered either good (BPM-P) or very good (BMP-T). This item was included in the BPM based on earlier research indicating high factor loadings in both population and clinical samples [55]. Although this item was within the acceptable range, one might consider that the participating children were young, i.e., aged between 8 and 12 years, which is the lower age range recommended for the use of the BPM [18]. The individual differences may be large in this age group, which might have had an impact on the scoring of this item. The two items, which were not present in the German study [24], contributed significantly to the factor of attention in both the BPM-P and BMP-T in the current study. For the EXT subscale, the factors loaded well on all items for both versions. The subscale INT showed larger differences between the factor loadings in the BPM-P than in the BMP-T. However, four items were considered excellent, and the item “feels too guilty” was considered very good, whereas “self-conscious or easily embarrassed” also reflected good factor loadings. The latter item had between a good and fair factor loading on the BPM-T, while the five other items showed excellent values. Self-consciousness and embarrassment might be more difficult to observe if the child does not express these feelings overtly. Children at this age, and especially children with internalizing problems, may have difficulties expressing their emotions. An alternative explanation could be that increased self-consciousness and embarrassment typically become a challenge at an older age, i.e., around puberty [56]. Nevertheless, when examining all the items in our analysis within each of the subscales, all factor loadings were high and significant. The suggested three-factor model of the BPM was confirmed, indicating that the instrument in our sample assessed what was intended, thus displaying strong construct validity.

The BPM has formerly been psychometrically evaluated in samples from the clinical and general population [21–24]. We add to existing knowledge by evaluating it in an “at-risk sample for internalizing problems”. This fits well to its use by school- or community services for children and adolescents to identify internalizing problems among children where one suspects such problems. Moreover, such a short early systematic assessment for children at risk would provide a reliable and valid base to prevent further development of anxiety or depression. It also could be a starting point for further referral to treatment in specialized mental health services.

### Strengths and limitations

One strength of this study was that it included a large heterogeneous sample of children from both rural and urban areas. The at-risk population of children presenting elevated symptom levels of anxiety and/or depression represents a sample less often studied than general population samples or clinical samples, which is also a strength of this study. A multi-informant approach with a high response rate, particularly from the teachers, together with sophisticated analyses, strengthened the results.

However, there are some limitations. The study was not initially designed to investigate the psychometric properties of BPM; therefore, data for tests of other instances of reliability (e.g., test–retest, sensitivity for change) and validity (e.g., convergent and divergent validity) were not available.

## Conclusion

The reliability of the Norwegian version of the BPM-P and BPM-T showed overall satisfactory internal consistency on all subscales and the total scale. Multi-informant agreement between the parents and the teacher reports were as expected—moderate on the externalizing, attention, and total scales, and low on the internalizing scale. Regarding validity, the original three-factor solution of the parents’ and teachers’ versions of the BPM was confirmed based on our sample of school children. Owing to the psychometric findings in this study, the BPM may be a valid, quick assessment tool for measuring attentional, behavioral, and internalizing problems in children. Further research in other Norwegian populations is needed to recommend the BPM for use in community health services. In addition, further evaluations that compare different short screening instruments developed during the last decade are desirable.

## Data Availability

The datasets generated and/or analyzed are not publicly available owing to privacy policy; however, they are available from the author on reasonable request.

## Abbreviations

ASEBA: Achenbach system of empirically based assessment
ATT: Attention problems
BPC: Brief problem checklist interview
BPM-T: Brief Problem Monitor-teacher form
BPM-P: Brief Problem Monitor-parent form
BPM-Y: Brief Problem Monitor-youth form
CBCL: Child behavior checklist
CI: Confidence interval
CFA: Confirmatory factor analyses
CFI: Comparative fit index
DF: Degrees of freedom
EXT: Externalizing problems
INT: Internalizing problems
MASC-C: Multidimensional anxiety scale for children
RMSEA: Root mean square error of approximation
SD: Standard deviation
SMFQ: Mood and feelings questionnaire–short version
TLI: Tucker and Lewis index
TRF: Teachers’ report form
YSR: Youth self-report
WLSMV: Weighted least square estimator with robust standard errors and mean- and variance-adjusted chi-square test statistics
χ^2^/*df*: Chi-square relative to its degree of freedom
